# Chemical Insights Into the Synthetic Chemistry of Quinazolines: Recent Advances

**DOI:** 10.3389/fchem.2020.594717

**Published:** 2021-01-14

**Authors:** Muhammad Faisal, Aamer Saeed

**Affiliations:** Department of Chemistry, Quaid-i-Azam University, Islamabad, Pakistan

**Keywords:** pyrimidine, bicyclic compounds, synthesis, green chemistry, quinazolines, quinazolinones

## Abstract

In medicinal chemistry, one of the most significant heterocyclic compounds are quinazolines, possessing broad range of biological properties such as anti-bacterial, anti-fungal, anti-HIV, anti-cancer, anti-inflammatory, and analgesic potencies. Owing to its numerous potential applications, in the past two decades, there is an increase in the importance of designing novel quinazolines, exploring promising routes to synthesize quinazolines, investigating different properties of quinazolines, and seeking for potential applications of quinazolines. The present review article describes synthesis of quinazolines *via* eco-friendly, mild, atom-efficient, multi-component synthetic strategies reported in the literature. The discussion is divided into different parts as per the key methods involved in the formation of quinazoline skeletons, aiming to provide readers an effective methodology to a better understanding. Consideration has been taken to cover the most recent references. Expectedly, the review will be advantageous in future research for synthesizing quinazolines and developing more promising synthetic approaches.

## Introduction

**Figure 1:**
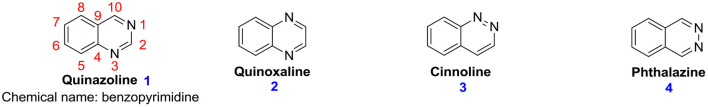
Structure of quinazoline and its isomers.

Quinazoline derivatives are among the most significant families of heterocyclic. Quinazoline (1,3-diazanaphthalene; **1**) is a moiety made up of two condensed six-membered aromatic rings, a pyrimidine ring, and a benzene ring (Wang and Gao, 2013). It is yellow and amorphous, and its molar mass is 130.15 g.mol^−1^, and the chemical formula is C_8_H_6_N_2_. On the basis of various substitution patterns of nitrogen atoms, it is isomeric with quinoxaline **2**, cinnoline **3**, and pthalazine **4** (Figure 1). These isomeric forms are also called diazanaphthalenes. Analogs of this family, which contain a pyrazine ring and a benzene ring, are called Quinoxaline **2**. These are also known as benzopyrazine. Cinnoline **3** also comprises a pyrazine ring and a benzene ring (Mishra, 2020). Phthalazine **4** is also called benzopyridiazine or benzo-orthodiazine, which contains a benzene ring and a pyridiazine ring. Gabriel (Ranawat et al., 2011) was the first scientist to prepare a quinazoline nucleus in the laboratory in 1903. Widdege (Asif, 2014) was the first scientist to propose the name quinazoline for this nucleus on the basis of its appearance as an isomer with the quinoxaline ring (Mahato et al., 2011). The synthesis of various compounds containing quinazoline as the main nucleus is largely mediated on the patterns of substitution on the 1,3-diazine entity of the system (Kamel et al., 2016).

**Figure 2:**
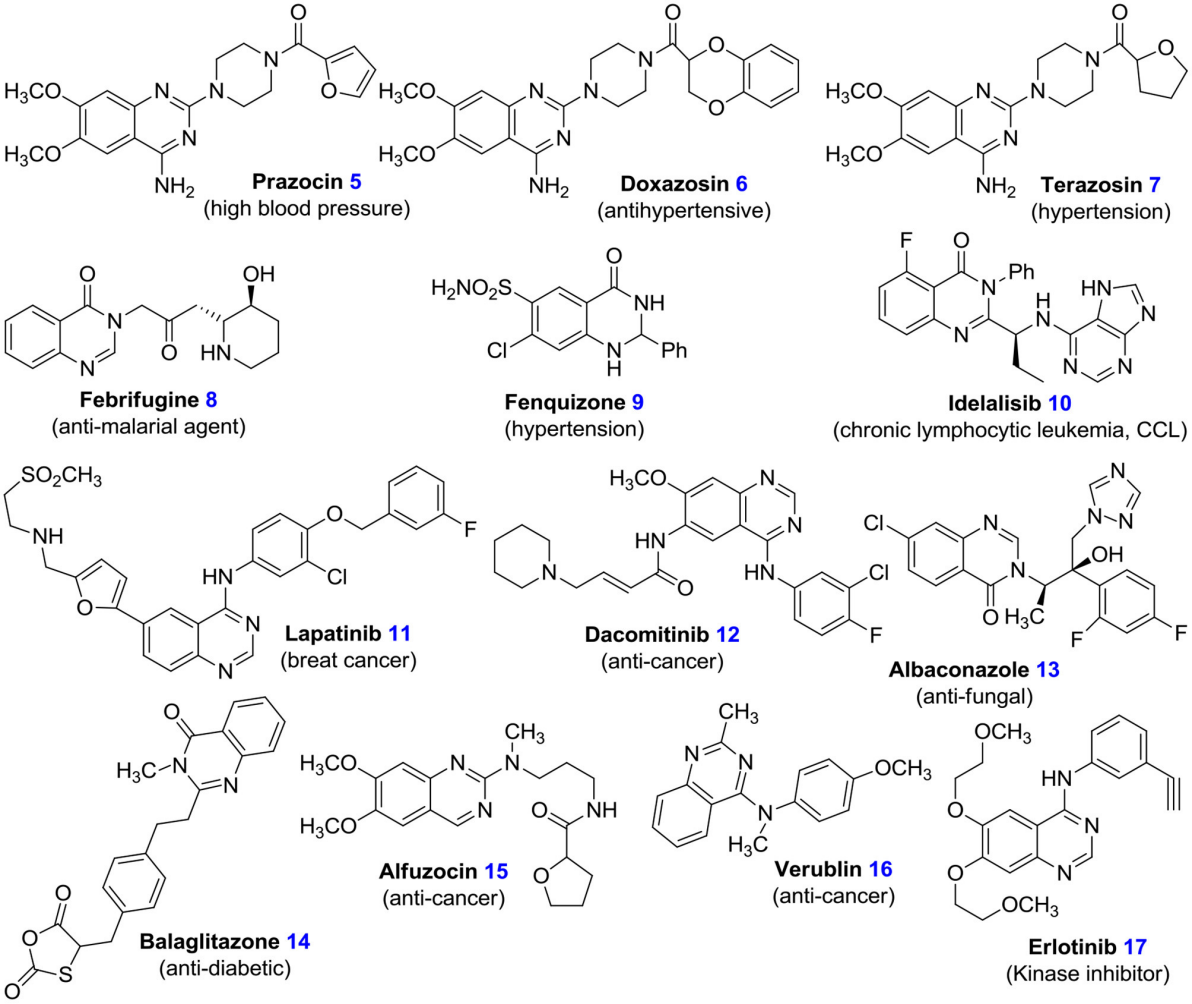
Structure of some quinazoline-based bioactive compounds.

Quinazolines are noteworthy in medicinal chemistry, on account of a wide range of their anti-viral (Alagarsamy et al., 2018), anti-HIV (Vijaychand et al., 2011), anti-malaria (Patel et al., 2017), anti-inflammatory (Karaman et al., 2008), anti-fungal (Alagarsamy et al., 2018), anti-bacterial (Bedi et al., 2004), anti-spasm (Wang and Gao, 2013), anti-cytotoxin (Mishra, 2020), anti-virus (Witt and Bergman, 2000), anti-analgesic (Selvam and Kumar, 2011; Kshirsagar, 2015), anti-cancer (Karaman et al., 2008), anti-oxidation (Iino et al., 2009), anti-hypertensive (Honkanen et al., 1983), anti-depressant (El-Sayed et al., 2019), anti-psychotic (Mizuno et al., 2010), anti-diabetes (Uckun et al., 2002), anti-tuberculosis activities (Kunes et al., 2000), and also their inhibitory effects on thyrosine kinase, poly-(ADP-ribose) polymerase (PARP), and thymidylate synthase (Eswaran et al., 2010). There are several approved drugs with quinazoline structure in the market such as, prazosin hydrochloride **5**, doxazosin mesylate **6**, and terazosin hydrochloride **7** (Figure 2) (Jafari et al., 2016; Devi et al., 2017). Also, many quinazoline derivatives act as DNA-binding agents or as effective adrenergic blockers (Kamel et al., 2016). Many quinazoline derivatives also constitute the building blocks for about 150 natural alkaloids isolated from numerous families of the plant kingdom, from animals, and from microorganisms. Earlier studies conducted in the 1950s and 1960s led to the discovery of febrifugine **8**, a quinazolinone-based alkaloid, which possesses anti-malarial potential, from the Chinese plant aseru (Wattanapiromsakul et al., 2003). Various quinazolinone-mediated drugs, which include fenquizone **9** and idelalisib **10**, have been observed to display a wide range of anti-fungal, anti-tumor, anti-microbial, and cytotoxic potencies (Witt and Bergman, 2000). In combination therapy, lapatinib **11** has been shown to be active for breast cancer (McKee et al., 1947). Dacomitinib **12** is used to treat NSCLC (non-small-cell lung carcinoma) (Kumar et al., 2009). Albaconazole **13** has potent and broad-spectrum anti-fungal activity. Balaglitazone **14** has been used in trials studying the treatment of diabetes mellitus. Alfuzocin **15**, verublin **16**, and erlotinib **17** are anti-cancer agents (Figure 2) (Selvam and Kumar, 2011; Kshirsagar, 2015).

Synthetic chemists prepared a library of quinazolines with different bioactivities by linking several active groups to the quinazoline entity using developing synthetic approaches, and the potential uses of the quinazolines in area of medicine, pesticides, and biology have also been disclosed (Khan et al., 2014, 2015). To be more precise, the position two, six, and eight of quinazoline nucleus is very significant for structural-activity investigations, and 2,3-difunctionalized quinazoline derivatives are observed to possess anti-viral, anti-hypertensive, and anti-bacterial functions (Bouley et al., 2016; Hrast et al., 2017). Incorporation of various heterocyclics, such as phenothiazine, triazole, and pyridine, at second position of quinazoline entity results in the development of insecticidal, anti-bacterial, and anti-fungal properties (McKee et al., 1947). The substitution of heteroaryl and aryl moieties at N-3 and C-2, respectively, has shown improved analagesic and anti-inflammatory activities (Iino et al., 2009). Substitution at second and third position, like bridge phenyl ring, phenyl ring, and heterocyclic rings, are shown to contain anti-microbial potency. Development of the lipophilic character at the C-4 position of the quinazoline ring would be desired for novel inhibitory affinity. Deactivating functional groups in the third position provides enhanced hypotensive efficacy. A phenyl ring at the eighth position and a nitro group at the sixth position of quinazoline entity possess improved anti-cancer potency. Quinazoline derivatives when bonded to thiadiazole ring bear anti-HIV, anti-fungal, and antibacterial activities. Incorporation of a stryl group at the second position in quinazoline (=O) leads to development of enhanced chemotherapeutic actions. Electron-rich substituents or halogens on the sixth position and substituted amine or simple amine on the fourth position are known to assist the potency against bacteria. The third position should be bonded to diverse heterocyclic rings for enhanced chemotherapeutic action (Kung et al., 1999; Bhattacharjee et al., 2004).

In the recent years, numerous synthetic approaches for the formation of quinazoline scaffolds have been disclosed (Rajput and Mishra, 2012; Srivastava and Srivastava, 2015; Hameed et al., 2018). This review delivers a broad picture of progress for the development of quinazolines over the last decade. To be more precise, the review consists of two parts. The first part provides transition metal-free approaches to afford quinazoline derivatives, including heterogeneous catalytic systems, microwave-assisted reactions, ionic liquid-based reactions, and visible light-mediated synthetic systems. The second part focuses on transition metal-catalyzed approaches to afford quinazoline derivatives, including ruthenium-, zinc-, rhodium-, cobalt-, nickel-, gold-, iron-, palladium-, and copper-catalyzed reactions to synthesize quinazolines. Hopefully, the literature review would be valuable for scientists working in the field of medicinal and synthetic chemistry.

## Transition Metal-Free Approach for the Synthesis of Quinazolines

In heterocyclic chemistry, transition metal-catalyzed coupling reactions have played a significant part for the formation of medicinally vital compounds. However, these reactions have some confronted challenges and limitation to some extent owing to the catalytic system. That is to say, most of the transition metals are toxic in nature, very expensive, and sensitive to moisture, especially oxygen. Also, the huge transition metal consumption does not meet the prerequisite for sustainable development. Alternative methodologies, therefore, for the development of C–N and C–C bonds under transition metal-free conditions are highly required and advantageous. In recent years, transition metal-free coupling reactions have become one of the attractive systems in synthesis to accomplish reactions with high productivity and to study how the reactions operate in the absence of transition metals.

### Microwave-Promoted Synthesis

**Scheme 1:**
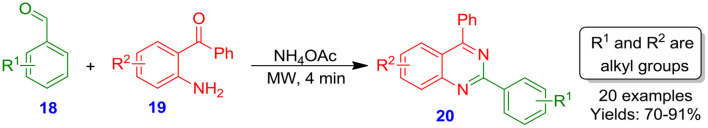
Reaction of aldehydes and 2-aminobenzophenones under microwave heating conditions.

Sarma and Prajapati reported a catalyst- and solvent-free synthesis of quinazoline derivatives **20** from aldehydes **18**, 2-aminobenzophenones **19**, and ammonium acetate under microwave heating conditions (Scheme 1). The presented protocol was equally operative with a diverse range of electron-deficient and electron-rich benzaldehydes **18**, and afforded target quinazolines **20** in good to excellent isolated yields (70–91%) within minutes (Sarma and Prajapati, 2011). The reaction is clean and simple, and provides an eco-friendly alternative toward removing organic solvents from organic synthesis.

### 4-Dimethylaminopyridine-Catalyzed Three-Component Approach

**Scheme 2:**
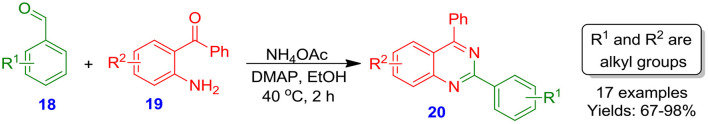
Reaction of aldehydes and 2-aminobenzophenones catalyzed by 4-dimethylaminopyridine (DMAP).

Boulcina et al. documented a general, efficient, one-pot process for the formation of quinazoline frameworks **20** in good to excellent isolated yields (67–98%) through the DMAP (4-[*N*,*N*-dimethylamino] pyridine)-catalyzed reaction of aromatic or hetero-aromatic aldehydes **18** with 2-aminobenzophenone **19** in the presence of NH_4_OAc under mild conditions (Scheme 2) (Derabli et al., 2014). This technique provides numerous benefits, for example, easy accessibility of starting materials, and high selectivity.

### Iodine/Ammonium Acetate-Assisted Three-Component Methodology

**Scheme 3:**
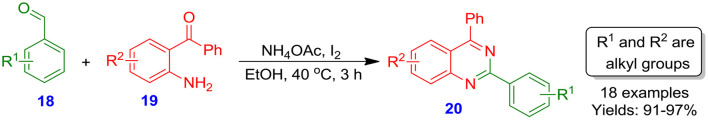
Iodine/ammonium acetate-assisted reaction of benzaldehydes with *o*-aminoarylketones.

Panja et al. described a three-component one-pot methodology for the synthesis of highly substituted quinazoline derivatives **20** (**34**) *via* I_2_-catalyzed reaction of substituted benzaldehydes **18** with substituted *o*-aminoarylketones **19** in the presence of NH_4_OAc (Scheme 3). When performed in neat or with EtOH even at moderate temperature, the reaction results in an excellent yield (91–97%) in lesser time. It was observed that iodine is the appropriate catalyst counterpart in this synthetic approach attributed to its oxidizing properties and Lewis acidity. Moreover, this technique is superior in terms of simplicity and non-involvement of chromatographic purification technique (Panja et al., 2012).

### Magnetic Ionic Liquid-Catalyzed Synthesis

**Scheme 4:**
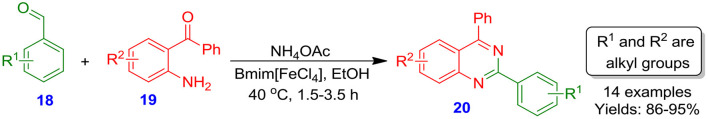
Reaction of benzaldehydes with *o*-aminoarylketones catalyzed by magnetic ionic liquid.

In another report, Panja et al. documented an ionic liquid (IL) Bmim[FeCl_4_] (butylmethylimidazolium tetrachloroferrate)-catalyzed one-pot, solvent-free, high yielding, multi-component green methodology for the synthesis of quinazolines **20** by the reaction of substituted aldehydes **18** with 2-aminobenzophenones **19** in the presence of NH_4_OAc at moderate temperature (Scheme 4). This technique was observed to be more valuable compared to other approaches in terms of its high catalyst stability, easier recyclability, high yield simplicity, and absence of any chromatographic purification technique (Panja and Saha, 2013).

### Base-Driven Synthesis in Water

**Scheme 5:**
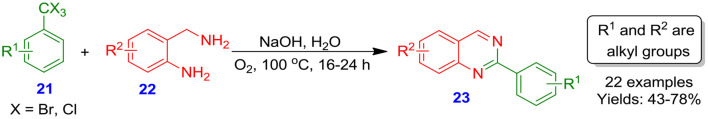
Reaction of α,α,α-trihalotoluenes and *o*-aminobenzylamines in water.

Cho et al. reported sustainable transition-metal-free synthesis of quinazoline derivatives **23** with moderate to good isolated yields (43–78%) from reaction of easily available α,α,α-trihalotoluenes **21** with *o*-aminobenzylamines **22** in the presence of molecular oxygen and sodium hydroxide in H_2_O (Scheme 5) (Chatterjee et al., 2018). The recrystallization process of the crude reaction mixture for the purification of the solid quinazolines eliminates the application of chromatographic purification and huge solvent-consuming workup, which make the overall process more economical and sustainable.

### Tetrabutylammonium Iodide-Catalyzed Amination

**Scheme 6:**
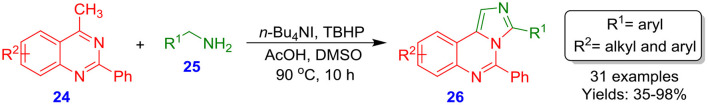
Reaction of benzylamines with 4-methyl-2-phenylquinazolines catalyzed by tetrabutylammonium iodide.

Li et al. disclosed tetrabutylammonium iodide (*n*-Bu_4_NI)-catalyzed tandem reaction for the formation of imidazo[1,5-*c*]-quinazolines **26** (Scheme 6). This technique was investigated by reacting various 4-methyl-2-phenylquinazolines **24** with benzylamines **25**, which afforded corresponding imidazo[1,5-*c*]quinazoline derivatives **26** with appropriate yields (35–98%) (Zhao et al., 2014). Selective dual amination of sp^3^ C-H bond under mild condition is involved in this reaction. Additionally, the reaction exhibited a wide range of substrates, including the common readily available α-amino acids and benzylamines. The novel procedure serves not only as a technique to develop a new series of imidazo-*N*-heterocycle derivatives **26** but also as a rare example of oxidative amination of benzylic primary C–H bonds with primary amines. This is a very valuable approach to transform simple quinazolines into highly functionalized quinazolines.

### Iodine/Tert Butylhydroperoxide-Driven C–H Functionalization

**Scheme 7:**
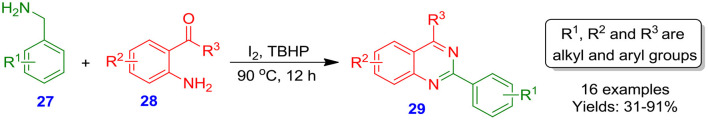
Reaction of benzylamines with 2-aminobenzophenones assisted by iodine/tert butylhydroperoxide (TBHP).

Zhang et al. documented an iodine/TBHP-assisted effective and facile one-pot tandem process for the development of 2-phenylquinazolines **29** with good to excellent isolated yields from benzylamines **27** and 2-aminobenzophenones **28** (Zhang et al., 2010). The method avoids the application of any kind of metal or hazard reagents (Scheme 7).

### Ceric Ammonium Nitrate-Catalyzed Synthesis

**Scheme 8:**
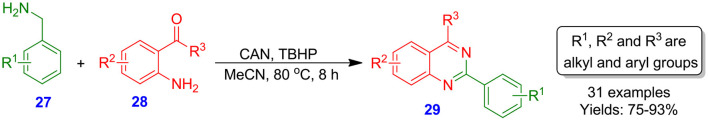
Reaction of benzylamines with 2-aminobenzophenones catalyzed by ceric ammonium nitrate (CAN).

Nageswar et al. demonstrated the construction of quinazoline scaffolds **29** with good to excellent isolated yields (75–93%) from reaction of benzylamines **27** and 2-aminobenzophenones **28** catalyzed by CAN/TBHP (ceric ammonium nitrate/*tert*-butylhydroperoxide) in CH_3_CN (Scheme 8). The CAN/TBHP system was observed to be efficient, mild, and novel reagent for the facile synthesis of quinazolines **29**. The yield of reaction was slightly increased when the electron-withdrawing group was present at the *para*-position of the benzylamine **27**, whereas an electron-donating group decreased the yield of product (Karnakar et al., 2011).

### 4-Hydroxy-TEMPO-Catalyzed C-H Bond Amination

**Scheme 9:**
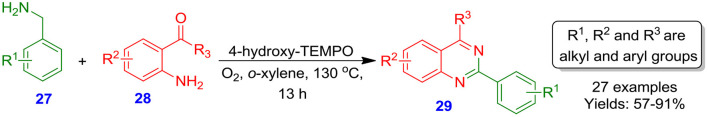
Reaction of benzylamines with 2-aminobenzophenones catalyzed by 4-hydroxy-TEMPO.

Han et al. described an effective and novel aerobic approach for the oxidative synthesis of 2-aryl quinazoline derivatives **29** *via* amination of benzyl C-H bonds using a one-pot 4-hydroxy-TEMPO radical-catalyzed reaction of 2-aminobenzaldehydes and 2-aminobenzoketones **28** with arylmethanamines **27**, without the use of any additives or metals (Scheme 9) (Han et al., 2011).

### Tert Butylhydroperoxide/DDQ-Induced Oxidative Cyclization Approach

**Scheme 10:**
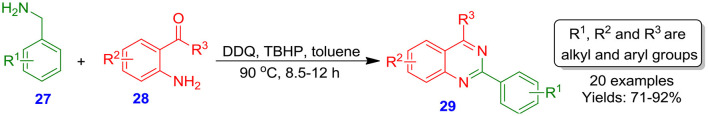
Reaction of benzylamines with 2-aminobenzophenones induced by tert butylhydroperoxide (TBHP)/DDQ.

Rachakonda et al. explored the synthesis of 2-arylquinazolines **29** under transition-metal-free and mild conditions from commercially available benzylamines **27** and 2-aminobenzophenones or 2-aminoacetophenones **28** *via* oxidative and condensation cyclization using DDQ as a versatile reagent (Scheme 10). The mechanism of the reaction involved condensation reaction followed by cyclization, giving the desired 2-arylquinazoline in good to excellent isolated yields (71–92%) (Rachakonda et al., 2012).

### Cobalt Zeolite Imidazolate Framework-Catalyzed Synthesis

**Scheme 11:**
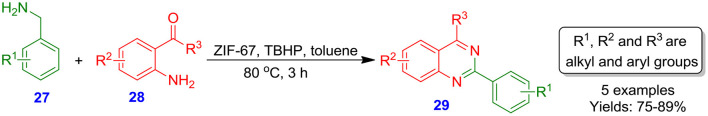
Reaction of 2-aminoacetophenones and benzylamines catalyzed by ZIF-67.

Truong et al. employed a heterogeneous catalytic system (*viz*. ZIF-67) for the cyclization reaction of benzylamines **27** with 2-aminobenzoketones **28** to afford quinazoline products **29** in excellent isolated yields (Scheme 11). Application of TBHP as an oxidant in toluene solvent at 80°C was observed to be at optimal conditions of reaction. ZIF-67 catalytic system could be regenerated and recycled without important degradation in catalytic potency (Truong et al., 2015).

### Molecular Iodine-Catalyzed C-H Bond Amination Using Oxygen as an Oxidant

**Scheme 12:**
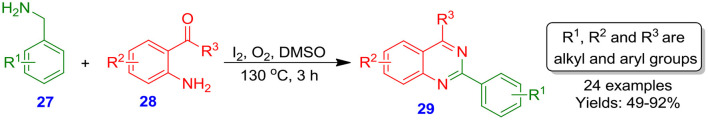
Reaction of benzyl-amines with 2-aminobenzophenones or 2-aminobenzaldehydes catalyzed by molecular iodine.

Very recently, Bhanage et al. reported the preparation of I_2_-catalyzed quinazoline derivatives **29** from reaction of 2-aminobenzaldehydes **28** or 2-aminobenzophenones with benzyl-amines **27** (Scheme 12). Numerous functionalized hetero-aryl or aryl amines were investigated with an ample range of functionalized 2-aminobenzaldehydes or 2-aminobenzophenones **28** to give the quinazolines **29** in moderate to excellent yields (49–92%) (Deshmukh and Bhanage, 2018). The application of O_2_ as an eco-friendly oxidant coupled with the solvent-, additive- and transition-metal-free conditions makes the approach greener and economical. The lack of aqueous workup also improves the productivity of this procedure. Moreover, the procedure uses I_2_ in catalytic amount and provides benzylic sp^3^ C–H bond functionalization/amination (Eswaran et al., 2010).

### Lewis Acid-Catalyzed Synthesis

**Scheme 13:**
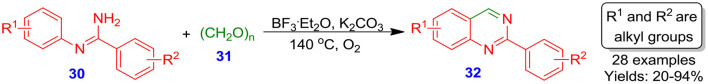
Reaction of *N*-phenyl-benzimidamides with paraformaldehyde catalyzed by Lewis acid.

In the recent years, Deng et al. reported an efficient method for the synthesis of quinazoline scaffolds **32** under transition-metal-free conditions from reaction of *N*-phenyl-benzimidamides **30** and polyoxymethylene **31** as one carbon source (Cheng et al., 2016). The optimized condition of reaction was well-tolerated with electron-deficient and electron-rich substituent on benzene ring with low to excellent yield of respective quinazoline derivatives **32** (20–94%) (Scheme 13). The transition-metal-free reaction and mild conditions are one of most attractive features of this technique. This novel process offers an easily handle-able, eco-friendly, and complementary approach to 2-arylquinazoline scaffolds.

### Iodine (III)-Driven Oxidative C–N and C–C Bond Construction

**Scheme 14:**
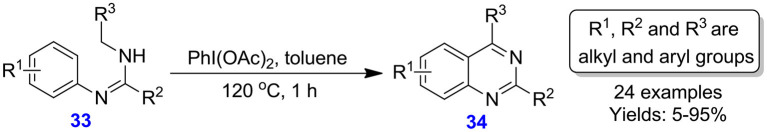
Cyclization of arylamidines promoted by iodine (III).

Lin et al. documented the preparation of multi-substituted quinazolines **34** from *N*-alkyl-*N*′-arylamidines **33** through the formation of iodine (III)-driven oxidative C(sp^2^)-N and C(sp^3^)-C(sp^2^) bonds under base- and metal-free conditions (Lin et al., 2014). Substrates having electron-deficient and electron-rich substituents on the aromatic ring afforded the respective quinazoline in low to excellent yields (5–95%); however, the reaction was incompatible with an aliphatic substituent (Scheme 14).

### Visible Light-Assisted Photo-Redox Oxidative Annulation

**Scheme 15:**
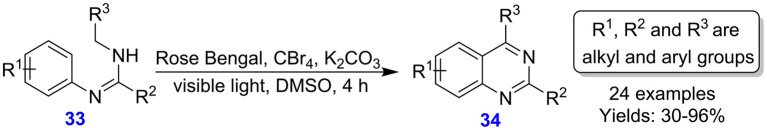
Cyclization of arylamidines catalyzed by photoredox organocatalyst.

Tang et al. reported the formation of quinazolines **34** from amidine derivatives **33** *via* formation of visible light-based oxidative C(sp^2^)–C(sp^3^) bond (Scheme 15). This synthesis is a metal-free oxidative coupling assisted by photo-redox catalytic system. The procedure features low loading of catalyst (1 mol %) (Shen et al., 2016). The approach was observed to tolerate a broad spectrum of functional groups.

### I_2_/KI-Based Oxidative C–C Bond Construction

**Scheme 16:**
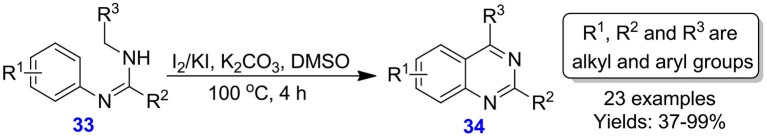
Cyclization of arylamidines catalyzed by I_2_/KI.

Lv et al. described the I_2_/KI-based oxidative C–C bond construction for the construction of quinazoline derivatives **34** from *N*,*N*′-difunctionalized amidines **33**. Under the standard condition, all *N*,*N*′-disubstituted amidines **33** converted into the corresponding quinazolines **34** in moderate to excellent yields (37–99%) (Scheme 16) (Lv et al., 2016). This environmentally benign and practical technique can also be performed on a gram scale and operates well with crude amidine precursors.

### Metal-Free Oxidative Annulation Using Cyanamide or Carbonitrile

**Scheme 17:**
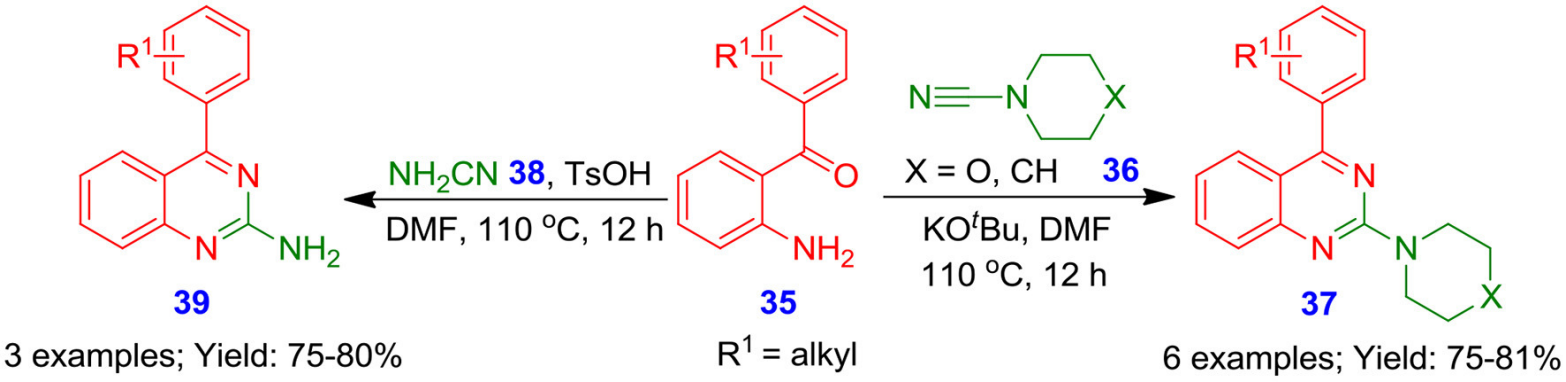
Reaction of 2-aminobenzophenones with 4-morpholinecarbonitrile or cyanamide.

North et al. demonstrated a promising methodology for the formation of 2-aminoquinazolines (**37**, **39**) in moderate to good isolated yields from the reaction of 2-aminobenzophenones **35** and 4-morpholinecarbonitrile **36** or cyanamide **38** (Pandya et al., 2017). The benefit of this synthetic approach is its transition-metal-free and mild conditions (Scheme 17). Of note, this process permits the synthesis of bioactive 2-aminoquinazoline analogous (**37**, **39**) using a cyclic amine or free amine, allowing good atom economy and structural diversity.

### Orthoester-Mediated Solvent- and Catalyst-Free Method

**Scheme 18:**
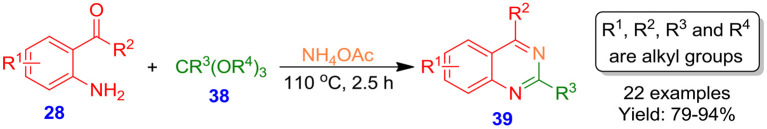
Reactions of 2-aminoarylketones with orthoesters under catalyst- and solvent-free conditions.

Bhat et al. disclosed a catalyst- and solvent-free environmental-friendly procedure for the formation of quinazoline frameworks **39** with good to excellent isolated yields (79–94%) from a one-pot three-component reaction of 2-aminoarylketones **28** and trialkyl orthoesters **38** in the presence of ammonium acetate (Scheme 18) (Bhat et al., 2015). The procedure bypasses some of the limitations and problems associated with the earlier techniques and is beneficial in terms of high product yield, simple work up procedure, moderate reaction time, readily availability of starting materials, clean reaction, and operational simplicity. Moreover, the protocol provides an environmental friendly and facile methodology toward the construction of bioactive novel quinazolines.

### Potassium Iodide-Promoted Three-Component Synthesis

**Scheme 19:**
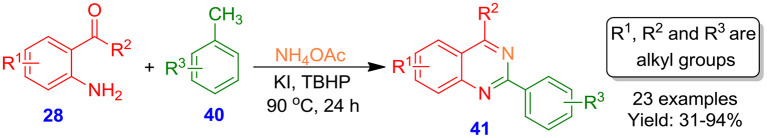
Reaction of 2-amino-benzophenones with methylarenes catalyzed by potassium iodide.

Li et al. synthesized 2-arylquinazoline derivatives **41** through a three-component one-pot mild KI-promoted methodology under transition-metal-free conditions by the reaction of 2-amino-benzophenones **28** with methylarenes **40** as one carbon source in the presence of NH_4_OAc (Zhao et al., 2015). The reaction demonstrated a wide spectrum of functional group tolerance including electron-deficient and electron-rich 2-aminoarylketones **28** and methylarenes **40** (Scheme 19).

## Transition Metal-Catalyzed Methodologies

Since the past century, transition metal-catalyzed C–H activation reactions have been investigated, and these reactions represent a promising achievement and development of organometallic chemistry. These reactions were started in 1960 as a key subject in organometallic chemistry and became one of the most effective catalytic systems for the formation of C–N and C–C bonds (Meijere and Diederich, 2004; Diederich and Stang, 2008). Moreover, transition metal-catalyzed C–H activation and functionalization have some advantages over classical technique such as straightforward method for the development of fused heterocyclic compounds, no need of pre-functionalization of starting material, which offers a more efficient reduction in the generation of waste. In the past decade, remarkable efforts have been made for the construction of heterocycles through the formation of C–N and C–C bonds. A brief summary of the recent literature of metal-catalyzed formation of quinazolines *via* C–H activation and C–N coupling reactions is described below.

### Palladium-Based Catalytic Systems

**Scheme 20:**
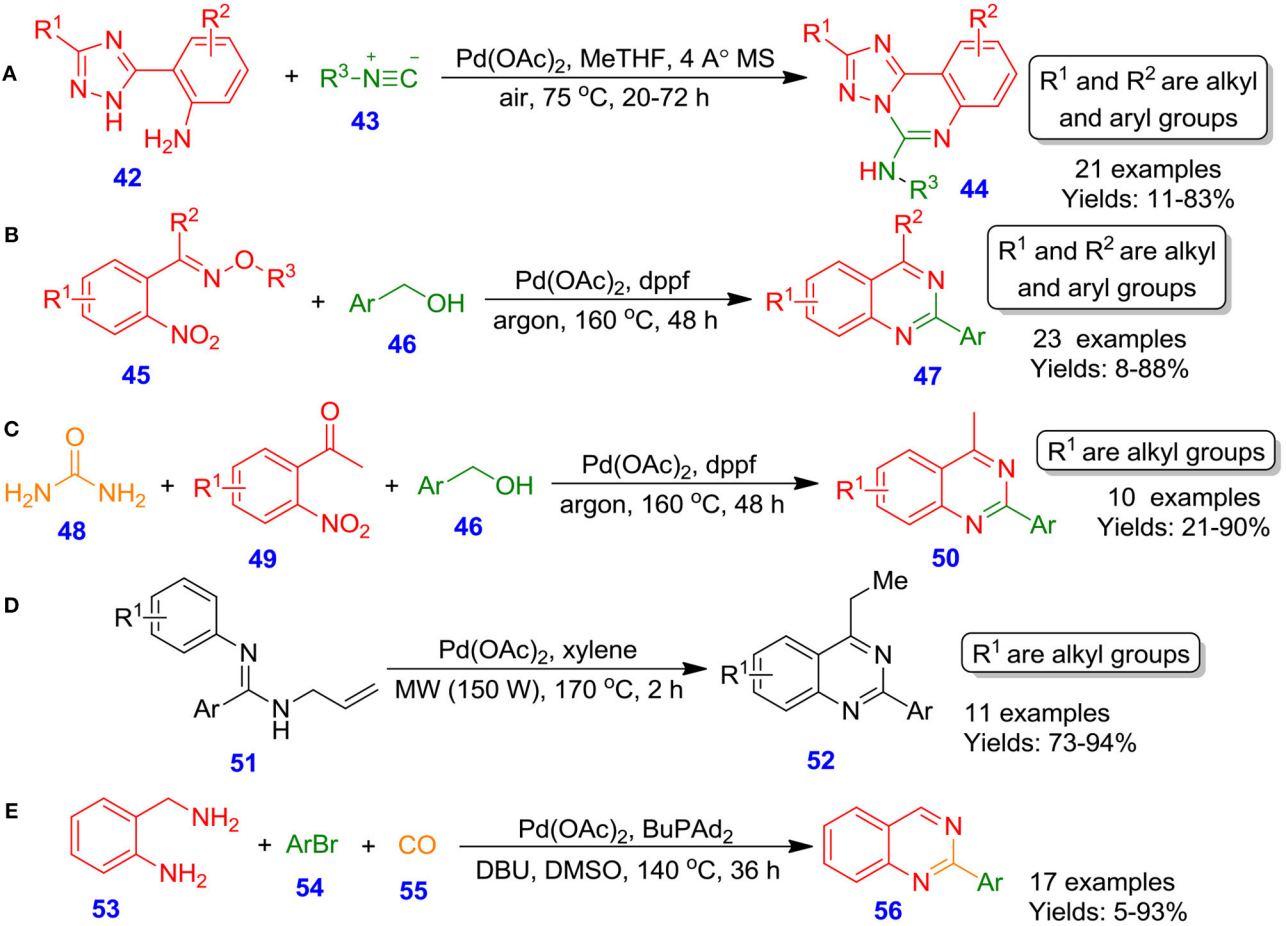
Pd-catalyzed preparation of diverse azolo-[c] quinazoline, 2-arylquinazoline and substituted quinazoline derivatives.

Vlaar et al. reported palladium(II) acetate-catalyzed aerobic oxidative coupling of (2-aminophenyl)azole derivatives **42** with isocyanide derivatives **43** for the formation of medicinally significant azole fused quinazolines **44** by using air as oxidant at 75°C in 2-methyltetrahydrofuran (Vlaar et al., 2014). An ample spectrum of triazole starting materials was reacted well and furnished annulated products in moderate to excellent isolated yields (11–83%) (Scheme 20A). In related development, Wang et al. described the palladium-catalyzed construction of 2-arylquinazoline scaffolds **47** by reacting *E*-1-(2′-nitrophenyl)ethanone*o*-methyloximes **45** and benzyl alcohols **46** in the presence of dppf as ligand under argon at 160°C through hydrogen transfer approach (Wang et al., 2014). It is supposed that the synthesis of quinazolines proceeds through the dehydrogenation of benzyl alcohols to benzaldehydes, followed by the formation of imine and subsequent intramolecular cyclization resulting in the development of quinazolines (Scheme 20B). Likewise, the reaction was performed out with benzyl alcohols **46**, urea **48**, and 1-(2-nitrophenyl)ethanone **49** under optimized condition. The efficient synthesis delivers quinazolines **50** in lower to excellent isolated yields (21–90%) (Scheme 20C). Similarly, Xu et al. described the annulation process for the formation of multi-substituted quinazolines **52** from *N*-allylamidines **51** under microwave heating conditions by employing palladium as an active catalyst in xylene at 170°C (Xu et al., 2015). The scope of this methodology was disclosed by using a spectrum of aryl amidines containing electron-deficient and electron-rich substituents, which provided the corresponding product in good to excellent isolated yields (73–94%) (Scheme 20D). In an interesting study, Chen et al. illustrated a novel and practical technique for the preparation of quinazoline scaffolds **56** from 2-aminobenzylamine **53** with aryl bromides **54** and carbon monoxide **55**, involving palladium-catalyzed aminocarbonylation–condensation–oxidation sequence and facilitating the desired quinazolines in poor to excellent isolated yields (5–93%) (Chen et al., 2014). In this procedure, DMSO serves both as oxidant and solvent (Scheme 20E). The generality of the approach was investigated by varying different withdrawing groups (cyano, trifluoromethyl) and electron releasing (methoxy, dimethylamino, or *tert*-butyl) containing aryl bromide.

**Scheme 21:**
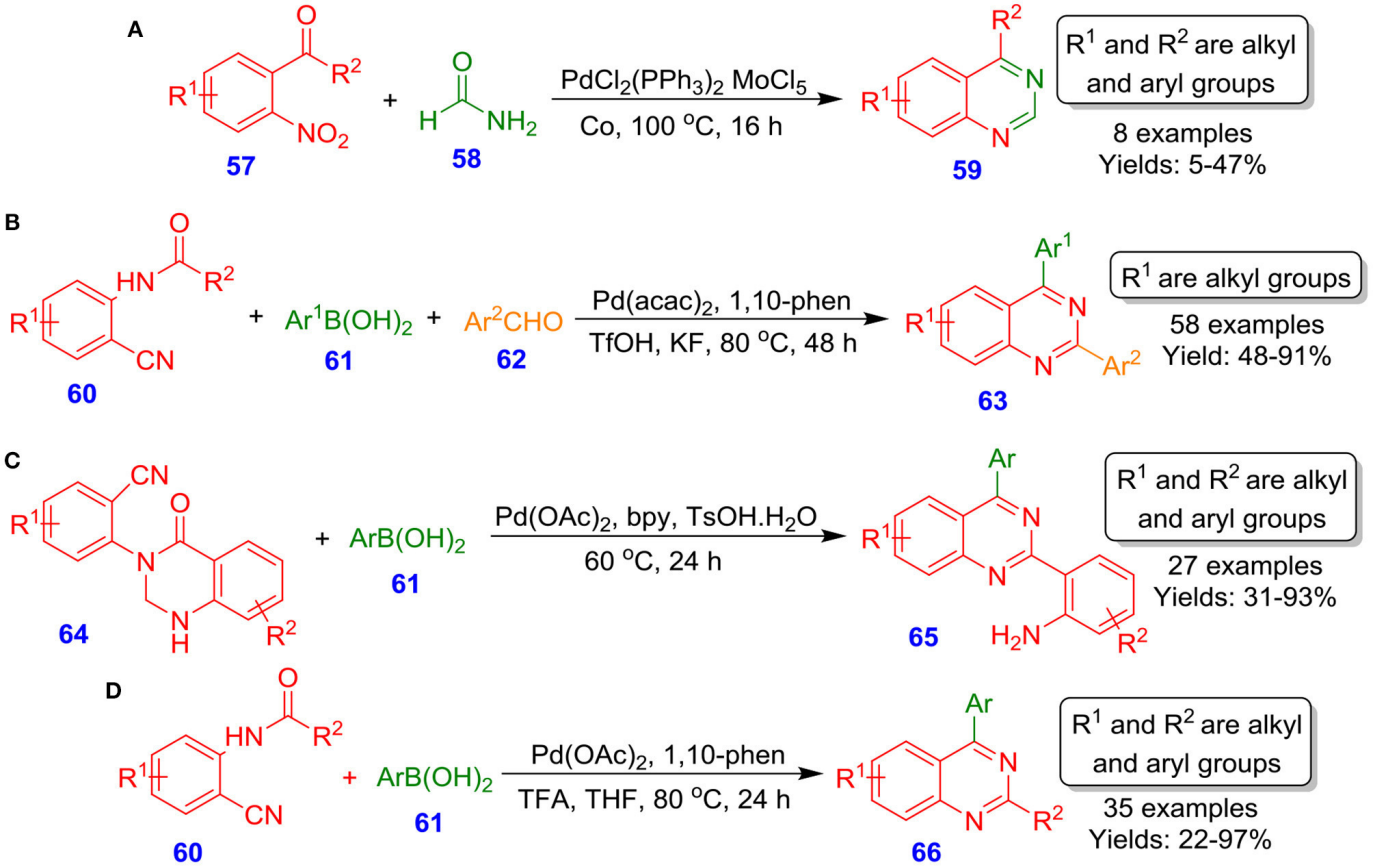
Palladium-catalyzed synthesis of quinazolinone-based nitriles and multi-substituted quinazolines.

Watanabe et al. described MoCl_5_ and Pd(PPh_3_)Cl_2_ catalyzing intermolecular reductive *N*-hetero-cyclization reaction of 2-nitrophenyl ketones or 2-nitrobenzaldehyde **57** with methanamide **58** for the formation of quinazoline analogous **59** (Akazome et al., 1995). The reaction proceeds through the formation of an active imene intermediate by selective carbon monoxide-based deoxygenation of nitro species (Scheme 21A). On the other hand, Chen et al. reported Pd-catalyzed three-component, one-pot tandem assembly for quinazolines **63** by using readily available 2-aminobenzonitriles **60**, aryl boronic acids **61**, and aldehydes **62**. The method displays broad substrate scope and amazing chemoselectivity (Scheme 21B). A notable feature of this technique is the tolerance of iodo and bromo moieties, affording flexibility for further synthetic manipulations (Hu et al., 2018). Later, the same research group disclosed another methodology for quinazoline scaffolds **65** from reaction of aryl boronic acids **61** with 2-(quinazolinone-3(4*H*)-yl)benzonitriles **64**. This tandem synthesis involved nucleophilic addition, followed by intramolecular cyclization and subsequent ring-opening, delivering the corresponding product in moderate to excellent isolated yields (31–93%) (Scheme 21C) (Zhang et al., 2018). In another interesting report by Chen et al., the synthesis of 2,4-disubstituted quinazoline derivatives **66** through Pd–catalyzed reaction of aryl boronic acids **61** with *N*-(2-cyanoaryl)benzamides **60** by employing 1,10-phen, trifluoroacetic acid in THF at 80°C has been described (Scheme 21D). The reaction revealed a wide spectrum of functional group tolerance, including electron-deficient and electron-rich aryl boronic acids **61** with *N*-(2-cyanoaryl)benzamides **60** (Zhu et al., 2018).

### Copper-Mediated Catalytic Systems

In literature, the efficiency of copper-based catalytic systems is well-documented since last century where these salts have demonstrated to be an effective catalytic system in cross-coupling reactions for the preparation of bioactive molecules and natural products, on account of their good functional group tolerance, low toxicity, and economic attractiveness (Deutsch et al., 2008; Allen et al., 2013; Guo X.-X. et al., 2015). Copper salts have become a promising alternative to their costly counterparts, for instance ruthenium-, rhodium-, and palladium-based catalytic systems for the cross-coupling reactions. Cu-catalyzed Ullmann reaction is the pioneering work in the area of synthetic organic chemistry (Sambiagio et al., 2014). Cu-catalyzed cross-coupling reaction has been broadly explored for the newer synthetic approach for fused heterocyclic entities.

**Scheme 22:**
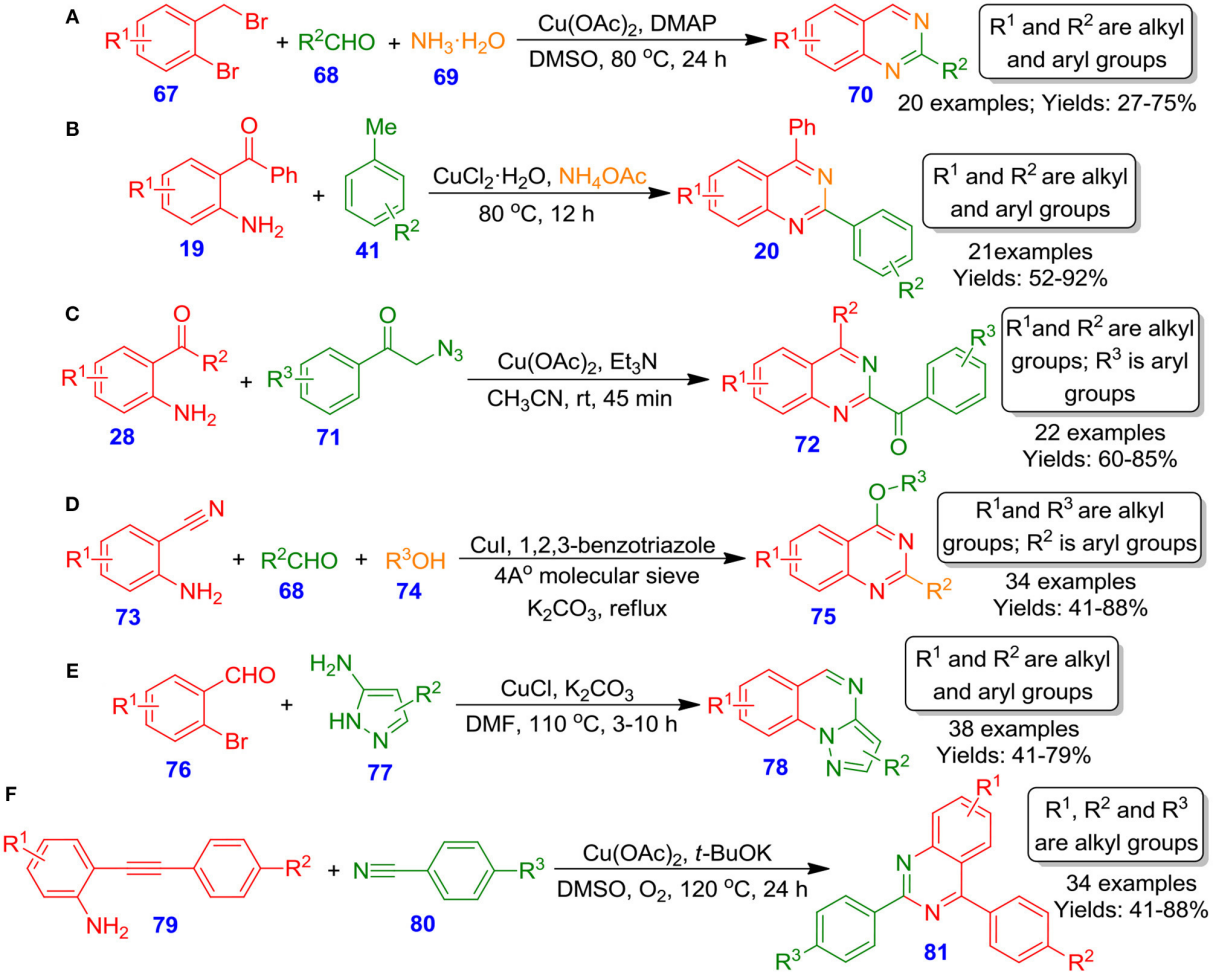
Copper–catalyzed synthesis of *o*-protected-4-hydroxyquinazolines, pyrazolo[1,5-a]quinazolines, tetrahydroquinazolines, 2-arylquinazolines, and multi-substituted quinazolines.

Fan et al. documented an effective one-pot method for the formation of diversely functionalized quinazolines **70** *via* copper-catalyzed tandem reaction among 2-bromobenzyl bromide derivatives **67**, aldehyde derivatives **68**, and ammonium hydroxide **69** (Fan et al., 2014). The reaction proceeds through cupric acetate-catalyzed amination of 2-bromobenzyl bromides **67** to 2-aminobenzyl amines, followed by condensation with aldehydes and subsequent intramolecular nucleophilic cyclization and aromatization, furnishing quinazoline derivatives (Scheme 22A). By employing simple aliphatic amines and ammonia as the source of nitrogen, the technique offers a practical and versatile approach. Also, this technique has several benefits, such as structural diversity of products and readily available starting materials. Alternatively, Liu et al. reported the direct approach to substituted quinazolines **20** from reaction among 2-aminobenzoketone derivatives **19**, toluene **41**, and ammonium acetate in the presence of copper(II) chloride at 80°C for 12 h (Liu et al., 2015). The reaction proceeds through oxidative amination of benzylic carbon–hydrogen bonds of methylarenes with 2-aminobenzoketones and ammonia, followed by intramolecular cyclization, affording quinazoline derivatives. Furthermore, the kinetic isotope effect (KIE) suggested that the carbon–hydrogen bond cleavage was the rate-limiting step in this methodology (Scheme 22B). On account of this method, a library of 2-arylquinazoline derivatives can be easily prepared in good isolated yields. Recently, in the same line, Kamal et al. employed 2-aminobenzophenones **28** and phenacyl azides **71** for the construction of quinazolines **72** by using cupric acetate, triethylamines in acetonitrile at ambient temperature (Visweswara Sastry et al., 2017). This procedure proceeded well and constructed two C–N bonds in a single operation (Scheme 22C). Additional, no oxidant or external source of nitrogen is demanded to accomplish the formation of quinazolines **72**. The process is practical for production of numerous functionalized quinazolines **72** with high functional group tolerance as well as a broad spectrum of substrates. On the same note, Vishwakarma et al. demonstrated Cu-catalyzed effective approach for forming *o*-protected-4-hydroxyquinazolines **75** from 2-aminobenzonitriles **73**, substituted aldehydes **68**, and substituted alcohols **74** through the development of an *N*-functionalized bicyclic precursor, followed by nucleophilic attack of the alkoxy moiety (Battula et al., 2014). The synthesis was sufficiently explored with a wide spectrum of substituted 2-aminobenzonitriles and aldehydes led to respective quinazolines **75** in moderate to excellent isolated yields (41–88%) (Scheme 22D). Subsequently, Gao et al. disclosed a one-pot tandem method for the efficient and straightforward preparation of pyrazolo[1,5-*a*]quinazolines **78** by treating 2-bromobenzaldehydes **76** with 5-aminopyrazoles **77** in the presence of potassium carbonate at 110°C through Cu-catalyzed imine creation followed by Ullmann type coupling resulting in fused quinazolines **78** (Scheme 22E). Diverse functionalized 5-aminopyrazoles and 2-bromobenzaldehydes tolerated well and provided respective quinazoline in moderate to good isolated yields (41–79%) (Gao et al., 2014). With benefits such as mild reaction conditions, simple synthetic procedures, and readily available starting materials, the technique developed could be considered as a promising technique. Further, very recently, Wang et al. published Cu-catalyzed one-pot process for the preparation of substituted quinazolines **81** by reaction of 2-ethynylanilines **79** with benzonitriles **80** using O_2_ as the sole oxidant (Wang et al., 2018). The reaction proceeded *via* effective cleavage of the carbon–carbon triple bond and formation of new carbon–nitrogen, and carbon–carbon bonds in a one-pot manner (Scheme 22F). Furthermore, the reaction showcased a broad spectrum of substituent tolerance with several 2-ethynylanilines and benzonitriles, offering an array of quinazoline derivatives in moderate to excellent isolated yields (41–88%). These quinazolines also exhibited good fluorescence quantum yield, aggregation-induced emission effect, and lifetime decay, enhancing the importance of quinazolines in material chemistry for future aspect.

**Scheme 23:**
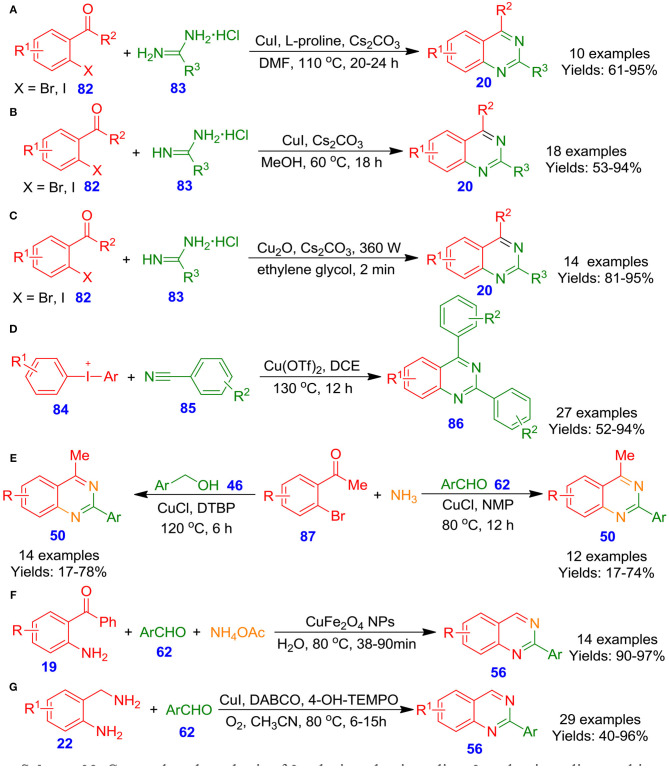
Cu–catalyzed synthesis of 2-substituted quinazoline, 2-aryl quinazoline, multi-substituted quinoline, and quinazolinone derivatives.

Fu et al. described quinazoline derivatives **20** *via* Cu-catalyzed tandem couplings of functionalized 2-halophenylketones or 2-halobenzaldehydes **82** with amidine hydrochlorides **83** under mild conditions (Huang et al., 2008). The approach was equally operative with aromatic as well as with aliphatic and delivered target quinazolines in good to excellent isolated yields (61–95%) (Scheme 23A). Moreover, the method showed simple, practical, and economical advantages. Similarly, Truong et al. reported an effective one-pot approach for highly functionalized quinazolines **20** in good to excellent isolated yields *via* ligand-free Cu-catalyzed Ullmann condensation of *o*-iodobenzaldehyde derivatives **82** with substituted amidine hydrochlorides **83** in the presence of Cs_2_CO_3_ in methanol at 60°C (Truong and Morrow, 2010). Mild reaction conditions as well as one-pot conditions make this procedure a striking alternative for the preparation of this family of compounds (Scheme 23B). In the recent years, Raut et al. achieved a facile and green ultrasonic-assisted formation of cuprous oxide nano-cubes as a heterogeneous nanocatalytic system at ambient temperature, and cuprous oxide nano-cubes were utilized for the construction of quinazoline frameworks **20** using one-pot tandem cyclization of 2-bromobenzaldehyde derivatives **82** with amidine hydrochlorides **83** without using any ligands (Scheme 23C). Numerous quinazoline derivatives **20** could be synthesized in excellent isolated yields within a few minutes. Additionally, the cuprous oxide nanocatalytic system could be regenerated and recycled up to four times without any important loss of catalytic potency (Raut et al., 2017). Along the same line, Wang et al. developed an effective one-pot procedure for the region-selective formation of functionalized quinazoline analogous **86** by using diaryl-λ^3^-iodanes **84** and nitriles **85** in the presence of cupric acetate and potassium *tert*-butoxide in dimethyl sulfoxide at 120°C (Wang et al., 2013). This approach of electrophilic annulations permits the use of commercially available materials and facilitates great flexibility of the substitution patterns on unsymmetrical or symmetrical diaryl-λ^3^-iodanes **84** and nitriles **85**, gave respective quinazolines **86** in good to excellent isolated yields (52–94%) (Scheme 23D). In another approach, Hua et al. disclosed cuprous chloride-catalyzed multicomponent one-pot formation of quinazoline analogous **50** by the reaction of *o*-bromo aromatic ketones **87** with aromatic aldehydes **62** or aromatic alcohols **46**, and ammonia in H_2_O (Ju et al., 2012). The most important features of this synthetic tool include good isolated yields and air or DTBP (when primary alcohols are used) as oxidants (Scheme 23E). In a related development, Farhang and Baghbanian documented an effective and eco-friendly one-pot formation of quinazolines **56** *via* magnetically isolable and recyclable CuFe_2_O_4_ nanoparticle catalyzed tandem cyclization reaction among aryl aldehydes **62**, 2-amino benzophenones **19**, and ammonium acetate (Baghbanian and Farhang, 2014). Nanoparticles of CuFe_2_O_4_ was easily synthesized by the thermal decomposition of Fe(NO_3_)_3_ and Cu(NO_3_)_2_ in H_2_O in the presence of NaOH (Scheme 23F). The catalytic potency of CuFe_2_O_4_ nanoparticles was investigated in aqueous media, revealing that this system is applicable as a promising, reusable, and green catalyst in organic synthesis. Moreover, the main benefits of the technique are (i) chemoselectivity, (ii) an insignificant loss of activity by using recycled catalyst, and (iii) simplicity in the extraction of the substrate/product from the catalysts. In the same connection, Han et al. explored a facile and effective one-pot reaction of 2-aminobenzylamine derivatives **22** with arylaldehyde derivatives **62** for the synthesis of quinazoline skeletons **56** by using DABCO/CuCl/4-HO-TEMPO as the catalytic system and oxygen as the terminal oxidizing agent (Han et al., 2012). Various substituted heteroaryl or aryl aldehydes **62** were treated with a variety of functionalized 2-aminobenzylamines **22** and furnished the functionalized quinazolines **56** in moderate to excellent isolated yields (40–96%) (Scheme 23G).

**Scheme 24:**
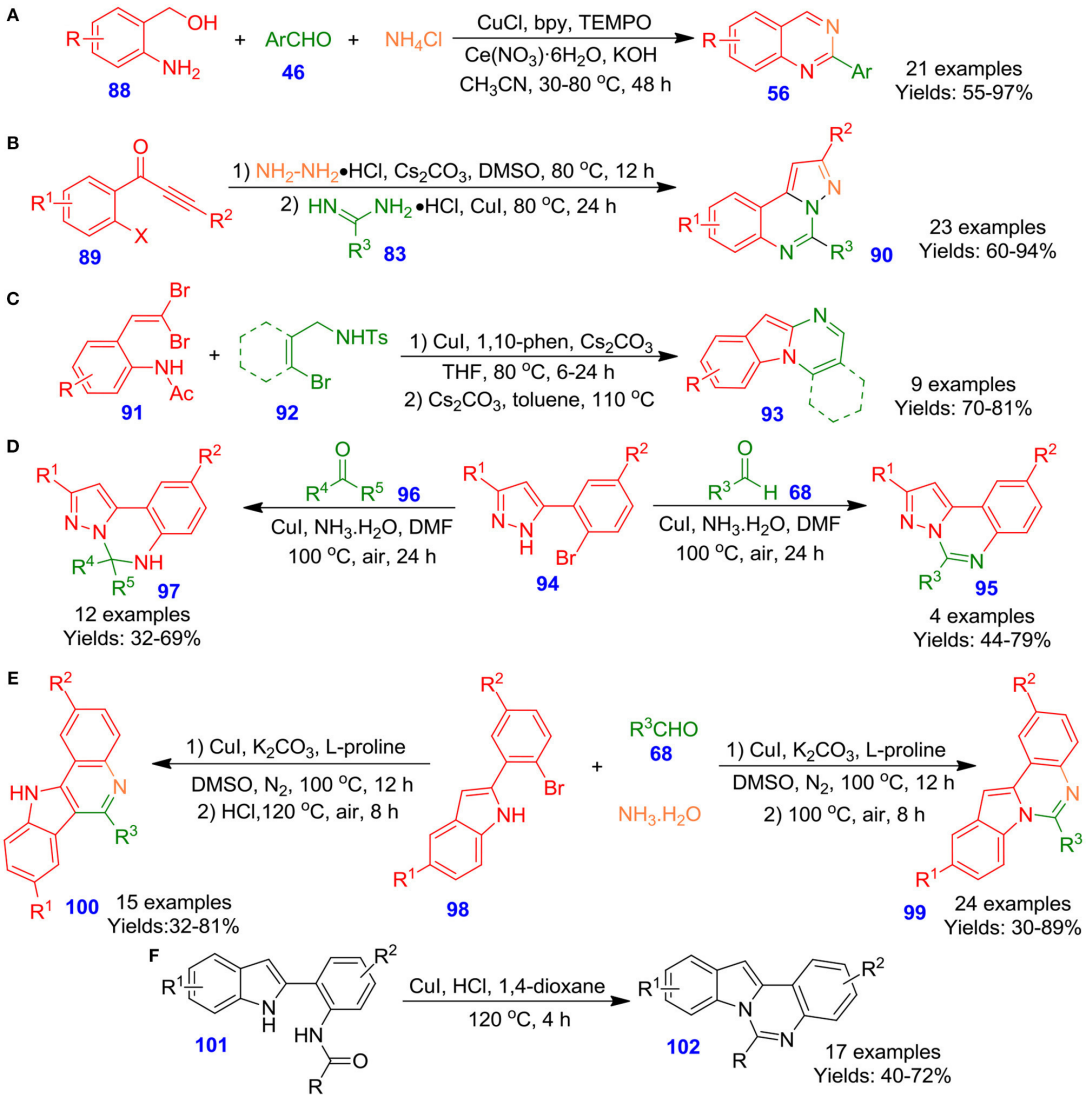
Cu–catalyzed synthesis of 2-aryl quinazoline, pyrazolo[1,5-c]quinazoline, and indolo[1,2-a]quinazolines, 2-(2-bromoaryl)-1*H*-indole derivatives.

Wu et al. reported the Cu-catalyzed one-pot tandem reaction among aldehydes **46**, (2-aminophenyl)methanols **88** and NH_4_Cl in the presence of TEMPO and cerium trinitrate at 80°C for 24 h. The technique represents a practical and convenient strategy for the formation of 2-functionalized quinazolines **56** (Scheme 24A). The reaction mechanism involved functionalization of 2-aminobenzylalcohols to 2-aminobenzaldehydes by using CuCl/TEMPO/2,2′-bipyridine(bpy) catalytic system. Following, the reaction of 2-aminobenzaldehydes with aldehydes and ammonium chloride afforded cyclized entity dihydroquinazolines, which on aromatization result in the formation of quinazoline frameworks in moderate to excellent isolated yields (55–97%) (Chen et al., 2013). Likewise, Yang et al. documented an efficient and novel process for the production of pyrazolo[1,5-*c*]quinazoline derivatives **90** *via* two-step one-pot reactions of commercially available functionalized 1-(2-halophenyl)-3-akylprop-2-yn-1-one derivatives **89**, amidine hydrochlorides **83**, and hydrazine hydrochloride under mild conditions, and the respective pyrazolo[1,5-c]quinazoline derivatives **90** were achieved in good to excellent isolated yields (Scheme 24B). The unique process can offer useful and diverse *N*-fused heterocycles for medicinal chemistry and combinatorial chemistry (Yang et al., 2012). In an interesting study, Kiruthika and Perumal disclosed a copper-catalyzed one-pot, intermolecular procedure for the rapid construction of indolo[1,2-*a*]quinazoline derivatives **93** from the commercially available *gem*-dibromovinylanilide derivatives **91** and *N*-tosyl-*o*-bromobenzamide derivatives **92** by employing Cs_2_CO_3_ and 1,10-phen in refluxing THF, followed by refluxing under basic conditions (Kiruthika and Perumal, 2014). The protocol operated well with a diverse range of *N*-tosyl-*o*bromobenzamide derivatives **92** and converted into respective quinazolines with good isolated yields (70–81%) (Scheme 24C). Moreover, this technique is practical, economical, and more reliable in terms of scalability, yield, and time. Keeping this in view, Gou et al. illustrated an effective technique for the development of pyrazolo[1,5-*c*]quinazolines **95** and 5,6-dihydropyrazolo[1,5-*c*]quinazoline derivatives **97** *via* one-pot Cu–catalyzed tandem reaction of 5-(2-bromoaryl)-1*H*-pyrazole derivatives **94** with ketones **96** or aldehydes **68** in ammonium hydroxide under aerobic conditions (Guo et al., 2013). A diverse spectrum of ketones and aldehydes, including hetero-aryl, alkenyl, alkyl, and aryl underwent efficiently in this reaction conditions and furnished corresponding functionalized quinazolines in moderate to good isolated yields (32–79%) (Scheme 24D). This synthetic process has the benefits of inexpensive starting materials and reagents, simple operation process, and broad scope of substrates. In another report by Fan et al., copper-catalyzed two-step one-pot sequential reactions of 2-(2-bromoaryl)-1*H* indole derivatives **98** with substituted aldehydes **68**, and ammonium hydroxide for the selective preparation of indolo[1,2-*c*]quinazolines **99** and 11*H*-indolo[3,2-*c*]quinolones **100** has been described (Guo S. et al., 2015). The regioselectivity of synthesis was maintained by regulating the reaction conditions. When the reaction was performed under acidic conditions, carbon–carbon coupling was observed, leading to the formation of 11*H*-indolo[3,2-*c*]quinolones in good isolated yields (32–81%). Having said that, in the absence of acid, formation of indolo[1,2-*c*]quinazolines was observed in moderate to excellent isolated yields (30–89%) (Scheme 24E). The current procedure features simple operation procedures and easily controlled selectivity. Very recently, Fan et al. described Cu-catalyzed aerobic oxygenation of 2-(2-amidoaryl)-1*H*indoles **101**, followed by intramolecular cyclization reaction under acidic conditions, resulting in the construction of quinazolines **102** in moderate to good isolated yields (40–72%) (Scheme 24F) (Guo et al., 2018).

### Ruthenium-Mediated Catalytic System

**Scheme 25:**
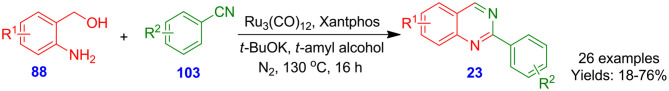
Ruthenium-catalyzed reaction of benzonitriles with 2-aminoaryl methanols.

Chen et al. explored straightforward Ru-catalyzed dehydrogenative synthetic protocol to afford 2-arylquinazoline derivatives **23** from the reaction of 2-aminoaryl methanol derivatives **88** with benzonitrile derivatives **103** in the presence of triruthenium dodecacarbonyl, potassium *tert*-butoxide, and Xantphos (Chen et al., 2015). A library of 2-aminoaryl methano derivatives **88** was successfully transformed in combination with various kinds of benzonitrile derivatives **103** into numerous desired quinazolines **23** in moderate to good isolated yields (18–76%) (Scheme 25). In this process, there is no need for the utilization of less eco-friendly halogenated substrates, providing a significant basis for constructing 2-arylquinazolines.

### Zinc-Based Catalytic System

**Scheme 26:**
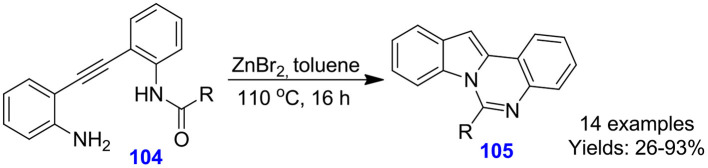
Zinc-catalyzed domino hydro-amination-cyclization for accessing quinazolines.

Wang et al. reported the zinc bromide (ZnBr_2_)-catalyzed domino hydro-amination cyclization approach for the development of indolo[1,2-*c*]quinazoline frameworks **105** from acyclic alkyne reactants **104** (Xu et al., 2013). The synthesis proceeds through ZnBr_2_-assisted tandem sequence, involving 5-*endo-dig* hydro-amination and intramolecular cyclization between an amide group with the indole nitrogen and gave indolo[1,2-*c*]quinazolines **105** in moderate to excellent isolated yields (26–93%) (Scheme 26). The method features mild condition as well as non-indole substrates, which are suitable for the forming of a panel of indolo[1,2-c]quinazolines.

### Rhodium-Mediated Catalytic Systems

**Scheme 27:**
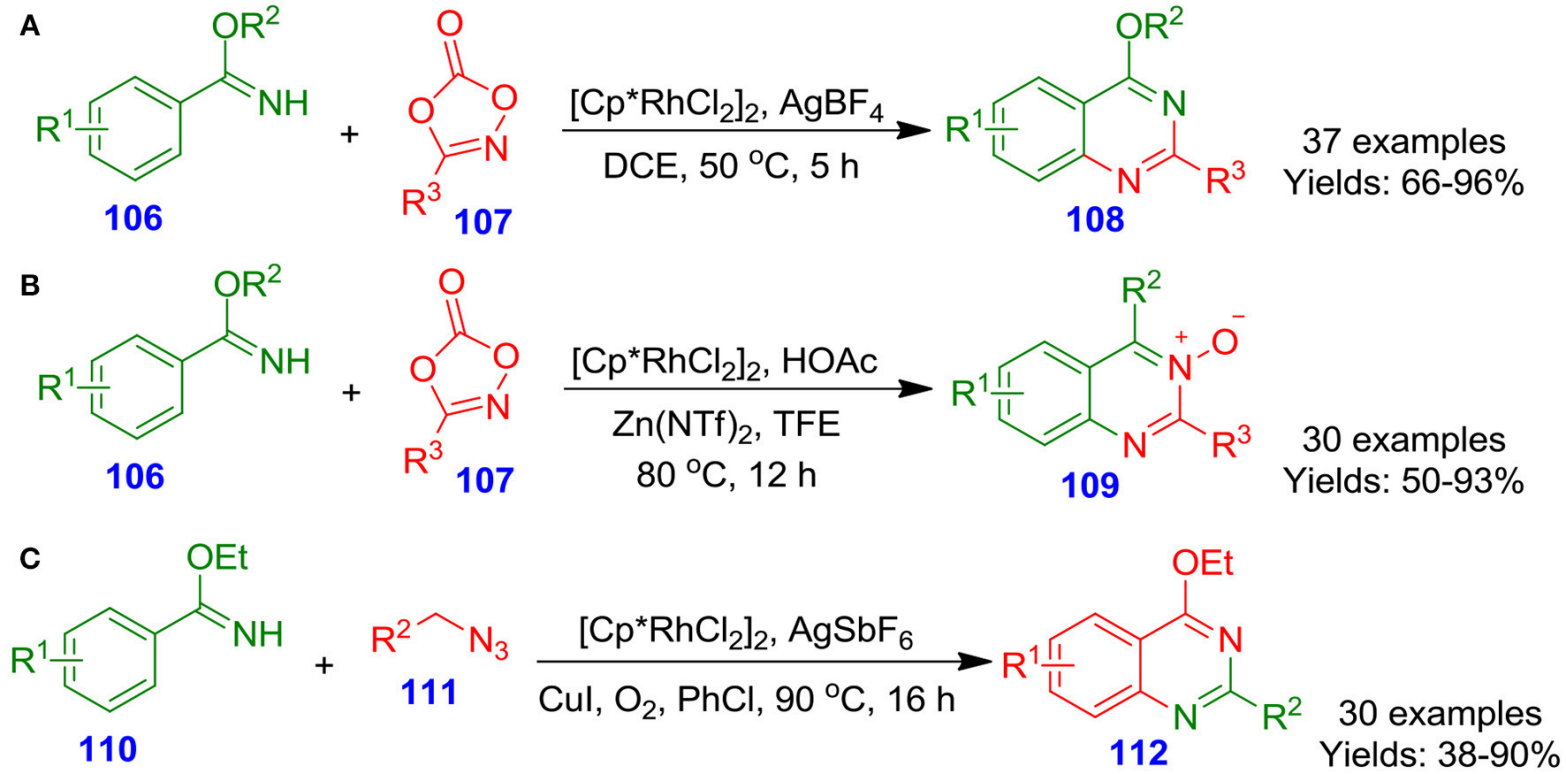
Various Rh-catalyzed synthetic methodologies for the formation of quinazoline skeletons.

Zhu et al. described the [Cp^*^RhCl_2_]_2_/AgBF_4_-catalyzed double carbon–nitrogen bond formation sequence for the construction of highly functionalized quinazolines **108** from reaction of benzimidate derivatives **106** with dioxazolone derivatives **107** (Wang J. et al., 2016). In this reaction, dioxazolone derivatives **107** also functioned as an internal oxidizing agent to regulate the catalytic cycle. A library of benzimidate derivatives **106** were transformed into corresponding quinazolines **108** with good to excellent isolated yields (66–96%) (Scheme 27A). The synthetic process proceeded with the benefits of operational simplicity, and high atom efficiency, and offered a significant basis for access to quinazoline derivatives. In the same year, Li et al. reported an effective synthetic procedure to access quinazoline *N*-oxides oxides **109** from ketoxime derivatives **106** and dioxazolon derivatives **107** through Zn(II)/Rh(III)-catalyzed carbon–hydrogen activation–amidation of the ketoxime derivatives (Wang Q. et al., 2016). This annulation tool proceeded effectively in the absence of any oxidant and delivered target quinazoline *N*-oxides oxides with good to excellent isolated yields (50–93%) (Scheme 27B). Furthermore, the reaction proceeded in high efficiency under mild conditions with water and carbon dioxide as the byproducts. Interestingly, Wang and Jiao documented an efficient and novel copper- and rhodium-co-catalyzed [4 + 2] carbon–hydrogen bond activation and annulation for the formation of biologically active quinazolines **112** from reaction of imidate derivatives **110** with alkyl azide derivatives **111** (Wang and Jiao, 2016). This aerobic oxidative procedure offers a valuable utilization of simple alkyl azide derivatives **111** in *N*-heterocycle synthesis with nitrogen and water as co-products (Scheme 27C). High atom efficiency and good functional group tolerance make this procedure suitable in accessing numerous functionalized quinazolines (Patel and Patel, 2019).

**Scheme 28:**
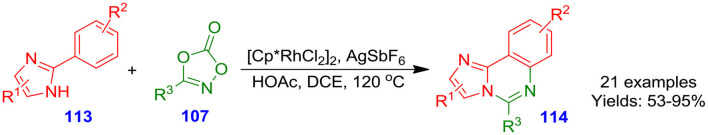
Rhodium-catalyzed annulation of carbon–hydrogen bonds.

In the recent years, Wu et al. described a rhodium-catalyzed direct and unique methodology for forming a library of 5-arylimidazo[1,2-c]quinazoline derivatives **114** in moderate to excellent isolated yields from annulation of ketones **107** and 2-arylimidazoles **113** (Scheme 28). This process is characterized by (i) free of halo functionalization handles; and (ii) commercially available starting material (Wu et al., 2018).

### Cobalt-Based Catalytic Systems

**Scheme 29:**
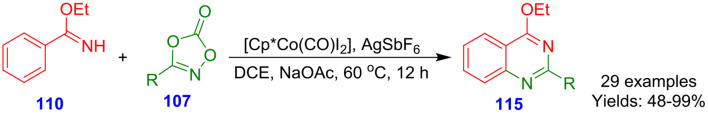
Co-catalyzed [4 + 2] cycloaddition of imine with dioxazolone.

Wang et al. developed cyclopentadienylcobalt dicarbonyl-catalyzed [4 + 2] cycloaddition of rarely explored dioxazolones **107** with imines **110** for the formation of multi-functionlized quinazolines **115** (Wang X. et al., 2016). The reaction involved cobalt-mediated tandem direct carbon–hydrogen amidation followed by intramolecular cyclization to deliver quinazolines with moderate to excellent isolated yields (48–99%) (Scheme 29). Cobalt-based catalytic system is exclusively suited to this conversion owing to its high sensitivity to steric hindrance and strong Lewis acidity.

**Scheme 30:**
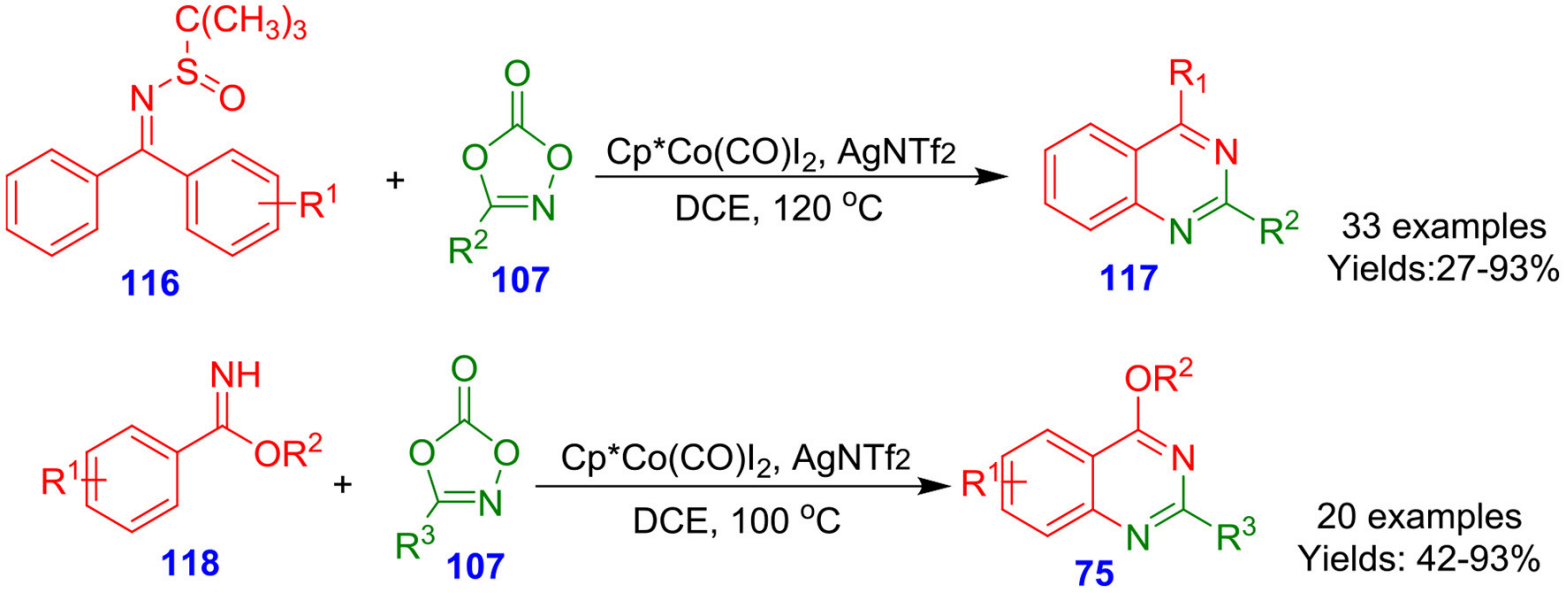
Cobalt-catalyzed activation of C–H bond for rapid formation of quinazolines.

Wang et al. documented cobalt-catalyzed direct functionalization of *N-*sulfinylimines **116** and benzimidates **118** with dioxazolones **107** for the rapid formation of quinazolines (**117** and **75**) (Scheme 30). Numerous dioxazolones **107**, benzimidates **118**, and *N*-sulfinylimines **116** actively contributed under the optimized reaction condition and afforded quinazolines in lower to excellent isolated yields (27–97%) (Wang F. et al., 2016). The synthesis of quinazolines proceeded with high mono-/di- and regioselectivity. In these synthetic tool, the dioxazolone coupling partners serve as a synthon of (the oxidized form of) nitriles.

**Scheme 31:**
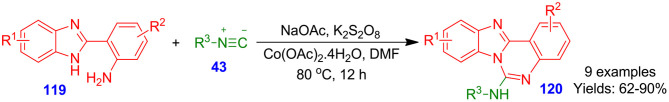
Co-catalyzed isocyanide insertion-cyclization for constructing benzoimidazoquinazoline frameworks.

In an interesting report, Ahmadi and Bazgir described a cobalt-assisted isonitrile insertion cyclization reaction for the formation of fused quinazoline frameworks **120**. To be more precise, treatment of isocyanides **43** with benzo[d]imidazol-anilines **119** in the presence of cobalt catalyst, sodium acetate, and potassium persulfate afforded quinazolines **120** (Ahmadi and Bazgir, 2016). The simple technique is highly useful, and it offers a straightforward methodology to a library of benzoimidazo-quinazoline amines (Scheme 31).

### Nickel-Mediated Catalytic Systems

**Scheme 32:**
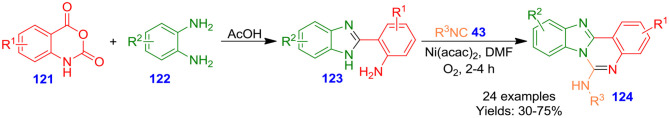
Ni-catalyzed sequential double annulation cascade (SDAC) approach to access fused quinazolines.

Sharada et al. described a ligand-base-free nickel-catalyzed one-pot sequential tandem approach for oxidative insertion of isonitrile under aerobic condition with intramolecular *bis*amine nucleophiles (Scheme 32). The tandem method involved an ring opening of isatoic anhydrides **121** followed by annulation to benzimidazoles **123** and subsequent nickel(II) *bis*(acetylacetonate)-catalyzed intramolecular insertion of isocyanide **43** result in fused quinazoline derivatives **124** with moderate to excellent isolated yields (30–75%) (Shinde et al., 2017). The base-/ligand-free features and application of dioxygen as the sole oxidant make this approach novel. The salient characteristics of this technique are the employment of inexpensive and commercially available starting materials, high bond-forming index (BFI), short reaction time, and the construction of four new carbon–nitrogen bonds in one pot fashion. Fluorescence investigation suggested that the synthesized quinazolines exhibit potent fluorescence properties with high quantum yield. These quinazolines have been proposed to be employed as a high fluorescent probe (Patel and Patel, 2019).

**Scheme 33:**
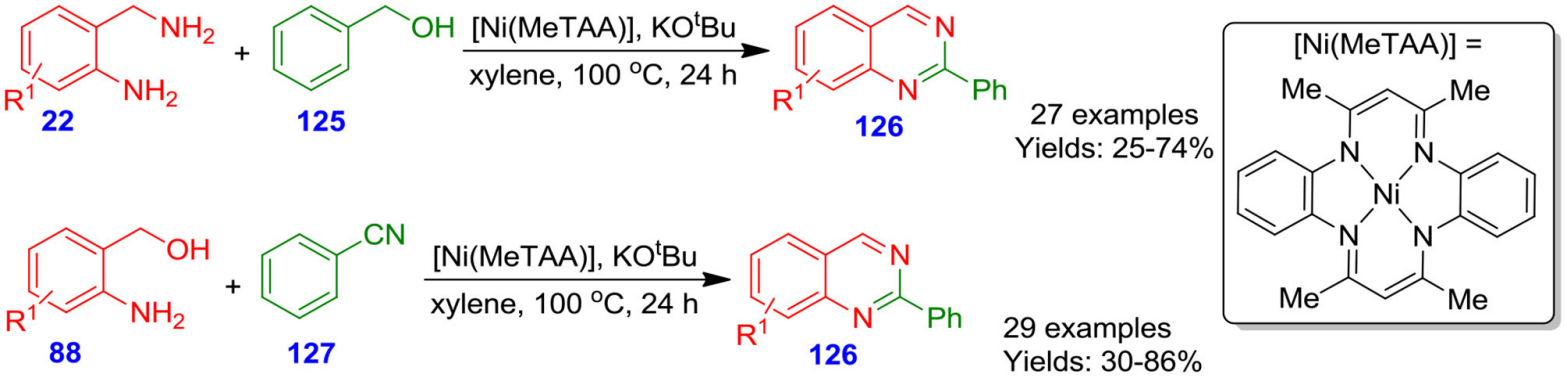
Ni-catalyzed acceptorless dehydrogenative coupling for accessing poly-substituted quinazolines.

Parua et al. developed nickel [Ni(MeTAA)]-catalyzed approach for the formation of quinazoline derivatives **126** from acceptor-less dehydrogenative coupling of 2-aminobenzylamines **22** with benzyl alcohols **125** and 2-aminobenzylalcohols **88** with benzonitriles **127** in the presence of potassium *tert*-butoxide in xylene at 100°C for 24 h (Parua et al., 2018). The environmentally benign methodology, easy to prepare nickel catalyst, and broad substrate scope made this methodology beneficial (Scheme 33).

### Gold-Based Catalytic Systems

**Scheme 34:**
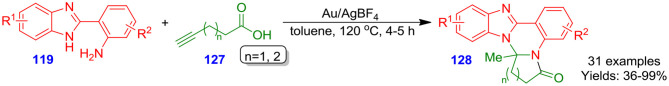
Au-catalyzed cascade method for the formation of quinazolinones.

Liu et al. described facile and efficient Ag(I)/Au(I)-catalyzed cascade technique for one-pot formation of benzo[4,5]imidazo[1,2]pyrrolo[1,2]quinazolinones **128** through the reaction of the functionalized 2-(1*H*-benzo[d]imidazol-2-yl)aniline derivatives **119** with 5-hexynoic acid or 4-pentynoic acid **127** (Ji et al., 2013). Furthermore, in the described procedure, substituent functionality in aniline derivatives **119** was generally well tolerated and gave respective quinazolinone derivatives **128** in moderate to excellent isolated yields (36–99%) (Scheme 34). The approach involved three new carbon–nitrogen bond formation in one-pot fashion.

**Scheme 35:**
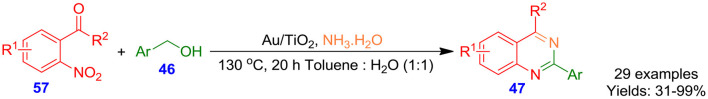
Ammonia-promoted and Au-catalyzed formation of quinazolines in water.

Alternatively, Wang et al. demonstrated hydrogen-transfer strategy for the preparation of 2,4-difunctionalized quinazolines **47** through a highly effective and selective nitrogen source-assisted reaction of aromatic alcohols **46** with *o*-nitroacetophenones **57** in the presence of Au/TiO_2_ as a catalytic system (Tang L. et al., 2015). The synthetic protocol is a wide substrate scope, has good tolerance to water and air, and signifies a novel avenue for economical and practical multiple carbon–nitrogen bond formation (Scheme 35). More significantly, no additional reductant, oxidant, and additive are demanded in the synthesis, and the catalytic system can be regenerated and recycled readily.

**Scheme 36:**
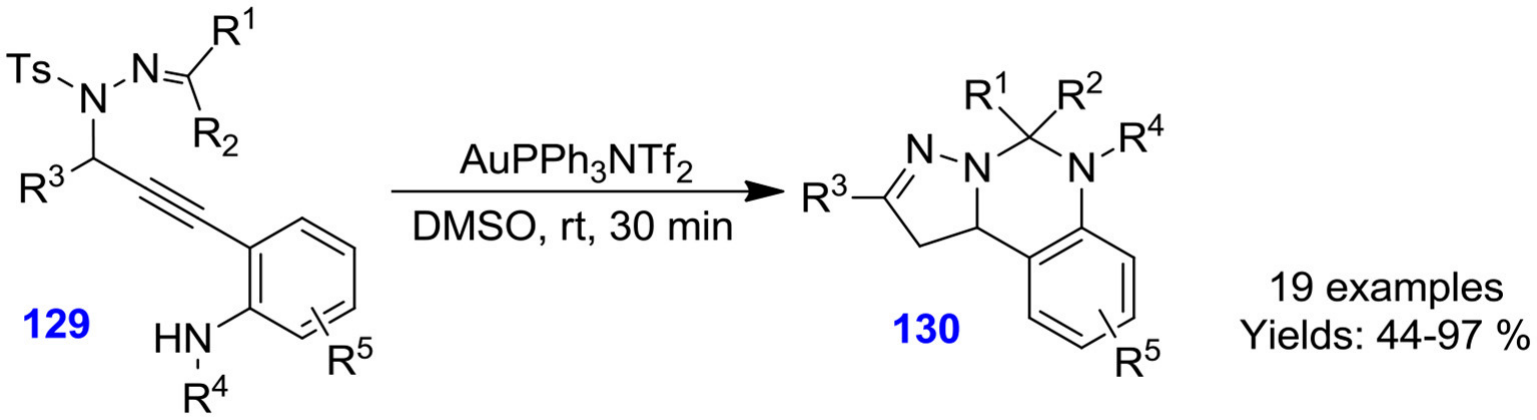
Au-promoted cyclization of *N*-propargylic sulfonyl hydrazones.

Tang et al. described gold-catalyzed chemo-selective cyclization of *N*-propargylic sulfonyl hydrazones **129** in dimethyl sulfoxide at ambient temperature for the development of 5,6-dihydropyrazolo[1,5-*c*]quinazoline derivatives **130** (Scheme 36). Numerous *N*-propargylic sulfonyl hydrazones actively converted under the optimized reaction condition to furnish quinazolines in good to excellent isolated yields (44–97%) (Tang H.-T. et al., 2015).

### Iron-Based Catalytic Systems

**Scheme 37:**
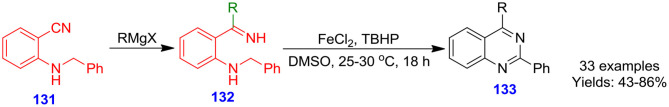
Fe-catalyzed oxidative amination of nitrogen–hydrogen ketimines.

Chen et al. reported ferrous chloride-catalyzed carbon–hydrogen oxidation and intramolecular carbon–nitrogen bond formation for the construction of quinazolines **133** using *tert*-butyl hydroperoxide as terminal oxidant. 2-Alkylamino N-H ketamines **132** were prepared *via* reaction of commercially available 2-alkylaminobenzonitrile **131** with Grignard reagent (Chen et al., 2018). The process delivered a broad variety of 2,4-difunctionalized quinazoline derivatives in good to excellent isolated yields (43–86%) (Scheme 37). The oxidation of the *N*-alkyl moiety in this procedure employs cheap and non-toxic iron salts (FeCl_2_) in the absence of any privileged ligands.

**Scheme 38:**
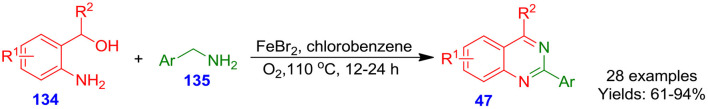
Ferrous bromide-catalyzed cascade reaction of benzylamines with 2-hydroxymethylanilines.

Ferrous bromide-catalyzed one-pot cascade approach for the preparation of quinazoline analogs **47** have been disclosed by Gopalaiah et al. from 2-hydroxymethylanilines **134** with aromatic amines **135** under an aerobic oxidative condition in benzene chloride at 110°C for 12–24 h (Gopalaiah et al., 2017). In a one-pot manner, the reaction proceeds through the construction of *N*-benzylidenebenzylamine intermediate and subsequent oxidative trapping of ammonia/intramolecular cyclization (Scheme 38). Both heteroaromatic/aromatic amines **135** treated smoothly in this approach and delivered corresponding quinazoline analogs **47** in good to excellent isolated yields (61–94%). This technique shows a wide substrate scope and is applicable to gram-scale synthesis. Moreover, the employment of molecular oxygen as an oxidant and an inexpensive and abundant iron salt as a catalyst makes this conversion very sustainable and practical (Patel and Patel, 2019).

**Scheme 39:**
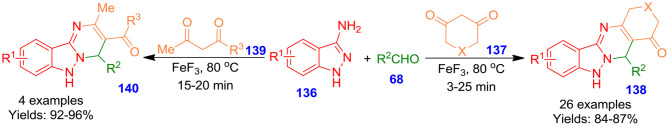
Sonochemical ferric fluoride-based synthesis of highly functionalized quinazolines under solvent-less conditions.

Jeong and Shinde described green and effective method for the construction of functionalized quinazoline derivatives (**138** and **140**) from indazol-3-amine derivatives **136**, aldehyde derivatives **68** and **137** or **139** catalyzed by ferric fluoride (FeF_3_) under ultrasonication in the absence of any solvent (Shinde and Jeong, 2016). The application of ultrasonication permits to accelerate the formation of a product from hours to only a few minutes, and ferric fluoride demonstrates excellent catalytic potency (Scheme 39). This effective green procedure offers amazing benefits such as easy work-up process, low cost, good to excellent yields, and eliminates the application of chromatographic purification. Also, the catalytic system can be easily regenerated and recycled for at least four runs without any important influence on the productivity of the quinazolines (Patel and Patel, 2019).

## Conclusion and Perspectives

In the review article, a broad spectrum of simple, mild, effective, novel synthetic routes to afford various functionalized quinazolines through cheap and commercially available starting materials have been reviewed. Clearly, a lot of work has been done for the construction of quinazoline frameworks in the recent past. Magnetic ionic liquid synthesis, nickel- and palladium-catalyzed synthesis, base-driven synthesis in water and microwave-promoted synthesis are highly advanced, novel approaches, with numerous positive aspects like mild reaction conditions, time efficient, recyclable catalysts, and use harmless solvents. Most importantly, several strategies including Lewis acid-catalyzed synthesis, cobalt zeolite imidazolate framework-catalyzed synthesis, CAN-catalyzed synthesis, base-driven synthesis in water, iron-catalyzed synthesis, and zinc-catalyzed synthesis have been successfully utilized to attain diversely decorated frameworks of quinazoline, which are important in agrochemical and pharmaceutical industries. The regularly improved synthetic approaches better the synthetic research on quinazolines with a tendency of faster, more convenient, and more diverse. Furthermore, because of the simplicity of synthetic methods enabling the construction of core scaffolds of many marketed drugs, we hope to see further research in the design of novel functionalized quinazoline derivatives, with exploitation of their biological activities in diverse ways.

## Author Contributions

All authors listed have made a substantial, direct and intellectual contribution to the work, and approved it for publication.

## Conflict of Interest

The authors declare that the research was conducted in the absence of any commercial or financial relationships that could be construed as a potential conflict of interest.
